# Accumulation of *cis*- and *trans*-regulatory variations is associated with phenotypic divergence of a complex trait between yeast species

**DOI:** 10.1093/g3journal/jkab016

**Published:** 2021-01-28

**Authors:** Offir Lupo, Gat Krieger, Felix Jonas, Naama Barkai

**Affiliations:** 1 Department of Molecular Genetics, Weizmann Institute of Science, Rehovot 76100, Israel; 2 Department of Plant and Environmental Sciences, Weizmann Institute of Science, Rehovot 76100, Israel

**Keywords:** gene regulation, genome evolution, transcription factors binding

## Abstract

Gene regulatory variations accumulate during evolution and alter gene expression. While the importance of expression variation in phenotypic evolution is well established, the molecular basis remains largely unknown. Here, we examine two closely related yeast species, *Saccharomyces cerevisiae* and *Saccharomyces paradoxus*, which show phenotypical differences in morphology and cell cycle progression when grown in the same environment. By profiling the cell cycle transcriptome and binding of key transcription factors (TFs) in the two species and their hybrid, we show that changes in expression levels and dynamics of oscillating genes are dominated by upstream *trans-*variations. We find that multiple cell cycle regulators show both *cis-* and *trans-*regulatory variations, which alters their expression in favor of the different cell cycle phenotypes. Moreover, we show that variations in the cell cycle TFs, Fkh1, and Fkh2 affect both the expression of target genes, and the binding specificity of an interacting TF, Ace2. Our study reveals how multiple variations accumulate and propagate through the gene regulatory network, alter TFs binding, contributing to phenotypic changes in cell cycle progression.

## Introduction

New phenotypes arise in evolution from mutations that change gene function or regulation. At the molecular level, the majority of mutations are neutral or of small phenotypic effect (Kimura 1968). Still, evolutionary-related species that express the same set of proteins differ in complex phenotypes, including size, growth pattern, and body morphology (King and Wilson 1975; Carroll 2000; Carroll 2008). Such phenotypic variation is often attributed to changes in regulatory sequences that alter gene expression, in either levels or spatial-temporal context. Supporting evidence was presented in the context of morphological traits, where interspecies differences in the positioning of body appendages or wing patterns were linked to variations in regulatory elements controlling the expression of major regulators, including central transcription factors (TFs) or signaling proteins (Carroll 1995; Brakefield *et al.* 1996; Keys *et al.* 1999; Jeong *et al.* 2006; Gomez *et al.* 2008; Martin *et al.* 2012).

Studying how gene expression evolves is therefore of major interest. Changes in gene expression can occur by mutations in local regulatory sequences (*cis*) or from upstream propagating variations (*trans*) (Hill *et al.* 2020). One way to distinguish between the two is by using an interspecific hybrid, in which the two genomes are exposed to the same *trans*-environment. Therefore, expression differences between orthologues genes in the hybrid are due to *cis*-effects, while interspecies differences that are buffered in the hybrid are due to *trans*-effects. Many studies have used the hybrid approach to characterize genome-wide expression variation in different species, revealing prevalent variation in gene expression both within and between species, and providing insights into the evolution of gene expression (Wittkopp *et al.* 2008; Tirosh *et al.* 2009; Emerson *et al.* 2010; Shi *et al.* 2012; Artieri and Fraser 2014; Combs *et al.* 2018; Krieger *et al.* 2020). How such variations manifest into phenotypes remains more elusive. This is of particular relevance to complex traits, which are often polygenic and inter-connected, confounding the link between expression variation and phenotypic variation (Boyle *et al.* 2017; Lee *et al.* 2019; Sella and Barton 2019).

As a model to address this question, we turned to budding yeast, which provides an experimentally accessible model for revealing the architecture of genetic variations associated with a complex trait. We examined two closely related yeast species, *Saccharomyces cerevisiae* (strain BY4743) and *Saccharomyces paradoxus* (strain CBS432), which exhibit phenotypic differences in morphology and cell cycle regulation when grown under the same environment (Herbst *et al.* 2017). Specifically, *S. paradoxus* cells are bigger, remain partially attached, and exhibit shorter G1 and longer G2 in comparison to *S. cerevisiae.* Studies in *S. cerevisiae* have extensively characterized the regulatory circuits controlling cell cycle progression, and identified numerous regulators that affect it and can therefore act as potential drivers of this altered growth phenotype (Morgan 2007).

Our goal in this study was to reveal the architecture of regulatory variation underlying the differences in cell cycle-related phenotypes between *S. cerevisiae* and *S. paradoxus*. By profiling the cell cycle transcription programs of the two species and the allele-specific expression of their hybrid, we found that central pathways regulating the cell cycle accumulated both *cis-* and *trans*-regulatory variations that bias the expression of regulators in the direction favoring the respected phenotype. These were found at multiple tiers; in main signal transduction components, in TFs and in their downstream target genes. Similarly, we found variations in cell cycle TFs that propagated through a combination of *cis-* and *trans-*effects to influence both the expression of target genes and promoter binding specificities of another TF. Specifically, regulatory variations affecting Fkh1 lead to downstream changes in the binding specificity of Ace2, a TF regulating G1 phase duration and cell separation. Our results suggest that phenotypic variation in complex traits, such as the cell cycle, occurs through distributed and propagating action of multiple regulators acting at different steps of a multi-layered response.

## Materials and methods

### Strains and plasmids

All strains used in this study and their genotypes are listed in Supplementary Table S1. For cell cycle experiments, strains used were *S. cerevisiae* of background BY4743 (Diploid) and *S. paradoxus* of background CBS432 (Diploid). The hybrid was created by mating *S. cerevisiae* Mat a with *S. paradoxus* Mat alpha.

For ChEC-seq experiments, each TF was C-terminally tagged with MNase. Yeast cells were transformed with the amplification product of MNase-Kanamycin cassette from pGZ108 plasmid, a gift from Steven Henikoff. Standard transformation using 50 bp homology-based recombination was used. Hybrids were created by mating haploid TF-MNase strain, both of *S. cerevisiae* and *S. paradoxus*, with WT haploids of the other species to create reciprocal hybrids, each expressing a TF-MNase allele of either species. ChEC-seq experiments on Ace2, Swi5, Fkh1, and Fkh2 were done on haploid strains. Ace2 and Swi5 were additionally checked in diploid strains (expressing one copy of TF-MNase) to control for ploidy effects.

Gene deletions were generated by replacing each relevant gene’s ORF by transformation and growth on plates containing selection. Strains of genotype *fkh1Δ*::hph and *fkh2Δ*::hph were generated by amplifying the hph gene from plasmid pBS35. Double deletion of genotype *fkh1Δfkh2Δ* was generated by amplifying the LEU2 gene from plasmid pRS425 and replacing FKH2’s ORF. Strains of genotype *ace2Δ*::KanMX and *swi5Δ*::KanMX were generated as previously reported (Krieger *et al.* 2020).

Swapped FKH2 strain was created in two steps: first, the FKH2’s ORF of *S. cerevisiae*, including terminator and promoter (150 bp downstream and 700 bp upstream, respectively), was replaced by an amplicon of the KanMX gene from plasmid pBS7. Second, a CRISPR-based method was used to induced break in the KanMX gene, while co-transforming with a genomic amplicon of FKH2 (including terminator and promoter) from *S. paradoxus*. Cas9 and the locus-specific 20 bp gRNA were expressed using plasmid bRA89. Ligation of the gene-specific gRNA into the bRA89 plasmid was done as previously described (DiCarlo *et al.* 2013).

All strains were validated by PCR and sequencing.

### Microscopy

Yeast cells were grown in YPD over night at 30°C to stationary phase and were inoculated to fresh medium for a few hours until reaching OD600 of 0.2. The cells were then prepared for imaging on YPD 2% low-melt agar pads in 96-well plate. Images were taken either in an Olympus IX83-based Live-Imaging system equipped with CSU-W1 spinning disc: sCMOS digital Scientific Grade Camera 4.2 MPixel Growth or Zeiss Axio Observer Z1 inverted microscope equipped with a motorized XY and Z stage, external excitation and emission filter wheels (Prior), IR-based Definite Autofocus from Zeiss and a 63× oil objective. The cells were kept at 30°C. Image adjustments and labeling were performed using imageJ.

### Time-course experiments

Yeast cells were grown in YPD over night at 30°C to stationary phase and were inoculated to fresh medium to OD600 of ∼0.005. When reaching an OD600 of 0.1–0.2, hydroxyurea (HU) was added to the media to a final concentration of 0.2 M for additional 2 h. To remove HU from the media, the cells were washed twice by centrifugation (4000 rpm for 1 min) and re-suspended in fresh, warm, equal-volume YPD. Then, the culture was returned to a bath orbital shaker. Cells were collected at the following time points: before HU (unsync), 5ʹ, 10ʹ, 20ʹ, 30ʹ, 60ʹ, and 120 min in HU, and every 5 min after release for 3 h. In total, 43 time points for each strain. For RNA, samples of 1.5 ml were taken and centrifuged for 10 s in 13,000 rpm, sup was removed and the pellets were immediately frozen in liquid nitrogen. For DNA staining, samples of 1.5 ml were taken and centrifuged for 10 s in 13,000 rpm and resuspended in cold 70% ethanol and kept in 4°C. This experiment was carried with two independent biological repeats for each strain.

### Flow cytometry—DNA staining

Cells were washed twice with 50 mM Tris-HCl pH8, re-suspended in RNase A for 40 min in 37°C, washed twice with 50 mM Tris-HCl pH8, and re-suspended in Proteinase K for 1-h incubation at 37°C. Then, cells were washed twice again, and re-suspended in SYBR green (1:1000) and incubated in the dark at room temperature for 1 h. Next, cells were washed from the stain, re-suspended in 50 mM Tris-HCl pH8 and sonicated in Diagenode bioruptor for 3 cycles of 10″ ON and 20″ OFF in low intensity. Cells were taken to FACS for analysis using BD LSRII system.

### RNA extraction and sequencing

RNA was extracted using a modified protocol of nucleospin^®^ 96 RNA kit (Machery-Nagel, cat 740466.4). Specifically, cells lysis was done in a 96 deep-well plate by adding 450 µl of lysis buffer containing 1 M sorbitol (Sigma-Aldrich), 100 mM EDTA, and 0.45 µl lyticase (10 IU/µl). The plate was incubated in 30°C for 30 min in order to break the cell wall, centrifuged for 10ʹ at 2500 rpm, and supernatant was removed. From this stage, extraction proceeded as in the protocol of nucleospin^®^ 96 RNA kit, only substituting β-mercaptoethanol with DTT. cDNA was prepared from the RNA extracts, barcoded, and sequenced using either Illumina HiSeq 2500 or Illumina NextSeq 500.

### Processing and analysis of RNAseq data

A pipeline for RNAseq data was created by Gil Hornung (INCPM, WIS). Fastq files were pre-processed by merging them into one file, removal of reads with high A or T content, trimming of the first three bases (usually GGG), and trimming of Illumina adapters and bases with quality <10 using cutadapt.

The reads were then mapped against a dual-species reference genome of *S. cerevisiae* and *S. paradoxus* strain CBS432 (Scannell *et al.* 2011). Genomes fasta and annotation files were downloaded from http://sss.genetics.wisc.edu/cgi-bin/s3.cgi. Mapping was performed with STAR 2.4.2a with the parameters—sjdbOverhang 60—scoreGap −10. The reads were divided based on the alignment scores. If for a read, the highest scoring alignment is assigned to a certain genome, and is unique in that genome, then it is assigned to that genome. If there is no difference in the scores between the two genomes and the alignment is unique in the cerevisiae genome, then the alignment to the cerevisiae genome is kept and saved as “indistinguishable.” Indistinguishable reads were discarded from further analysis. On average, 85% of aligned reads were mapped to either genome.

Counting was performed on the TES (Transcript End Site) region of each gene. The TES region is defined as 200 bp downstream from the end of the gene, and 500 bases upstream to the end of the gene. The reads were counted using htseq-count, with parameters—stranded yes and—mode union. Read counts per gene are found in Supplementary Table S2.

The sum of all reads in each sample was normalized to be 1,000,000 and genes with expression below a threshold of log2(10) in either species were excluded. All further data analysis was performed in Matlab.

### Expression of periodic genes

Periodic transcripts were defined by Cyclebase (Santos *et al.* 2015), and classified to groups based on Cyclebase expression peak time score. The top 500 Cyclebase genes were intersected with genes that were detectable in our experiment for both species, resulting in ∼430 periodic genes. For determining if a gene shows periodic expression, these 430 genes were classified into 12 groups based on expression peak time, the average expression along the timecourse of each group was calculated and the Pearson’s correlation between each gene to the average of its group was calculated. Significant correlations were those with corrected *P*-value < 0.05 (after Benjamini Hochberg FDR procedure), and rho >0.4.

### Differential expression analysis

Differential expression analysis was carried out using DESeq2 (Love *et al.* 2014) on R 3.6.3. Read counts were given as input to DESeq2 with the design: “∼ genotype,” where “genotype” differentiates the sample as coming from: cerevisiae, paradoxus, hybrid-cerevisiae, hybrid-paradoxus. This design enabled differential expression analysis between species and between hybrid alleles (*cis*-effect). Differential expression was determined via likelihood ratio test (test = “LRT”) focusing only on the interaction term for both designs, results went through log2 fold change (FC) shrinkage using ashr method (Stephens 2016). Significant differential expression were genes with *P*-value < 0.05 and log2(FC) >0.5.

### ChEC-Seq experiments

The experiments were performed as previously described (Zentner *et al.* 2015), with some modifications. Cultures were grown overnight to saturation in YPD media and diluted into 50 ml of fresh YPD media to reach OD_600_ of ∼0.3 the following morning after ∼10 divisions. Cultures were pelleted at 1500 g and resuspended in 1 ml Buffer A (15 mM Tris pH 7.5, 80 mM KCl, 0.1 mM EGTA, 0.2 mM spermine, 0.5 mM spermidine, 1 × Roche cOmplete EDTA-free protease inhibitors, 1 mM PMSF), and then transferred to DNA low-bind tubes (Eppendorf 022431021). Cells were washed twice more in 500 μl Buffer A, pelleted, and resuspended in 200 μl Buffer A containing 0.1% digitonin. Then, cells were transferred to an Eppendorf 96-well plate (Eppendorf 951020401) for permeabilization at 30°C for 5 min. CaCl_2_ was added to a final concentration of 2 mM for exactly 30 s for MNase activation. Next, 100 μl of stop buffer (400 mM NaCl, 20 mM EDTA, 4 mM EGTA and 1% SDS) were mixed with 100 μl sample. Proteinase K (100 μg, Sigma P2308) was then added, and samples were incubated at 55°C for 30 min. Nucleic acid extraction was performed as previously described (Zentner *et al.* 2015), with some modifications in the ethanol precipitation step; Samples were precipitated at −80°C for >1 h with 2.5 volumes of cold EtOH 96%, 45 μg Glycoblue (Thermo Fisher AM9515) and sodium acetate to a final concentration of 20 mM. DNA was centrifuged, washed with ethanol and treated with RNase A (Sigma, R4875) as previously described, followed by another round of DNA cleanup and ethanol precipitation. In order to enrich for small DNA fragments, reverse 0.8X SPRI clean-up was carried out. Library preparation was done as previously reported (Henikoff *et al.* 2011), except for the clean-up steps, which were performed using phenol-chloroform followed by ethanol precipitation as described above (instead of S400 columns). 1X SPRI was carried out on ChEC amplified libraries, which were then pooled and cleaned from adaptors dimers (150 bp) if needed. Libraries were sequenced on Illumina NextSeq500 for paired-end sequencing (50 bps for read1 and 15 or 25 bps for read 2).

Library construction for of ChEC-seq of Hybrid strain of FKH1-MNase and FKH2-MNase was slightly modified and phenol-chloroform clean-ups were replaced by SPRI-isopropanol clean-ups (Fishman *et al.* 2018).

All ChEC-seq experiments were repeated at least twice for each strain.

### ChEC-Seq processing and analysis

Reads were aligned using Bowtie2 (Langmead and Salzberg 2012) (parameters: –best –m 1) to a dual-species reference genome of *S. cerevisiae* R64 and *S. paradoxus* CBS432 (Yue *et al.* 2017). ChEC-Seq tracks, representing the enrichment of each TF, were calculated by adding +1 to each genomic location corresponding to the first nucleotide in a forward read, or the 50th position corresponding a reverse read. The signal was normalized to a total of 10 million reads, to control for sequencing depth. The median signal across repeats for each strain was taken for further analysis. For promoter analysis, promoters were defined only for genes with an annotated transcription start site (TSS) (Pelechano *et al.* 2013). The length of each promoter was defined as 700 bps upstream to the TSS. The signal across each promoter was summed to calculate overall promoter binding for each sample.

### Motif analysis

For the motif analysis, all possible x-mer sequences (5-mer for Ace2 and Swi5, 7-mer for Fkh1 and Fkh2) were given a numerical index (4^x/2 in total; forward and reverse complement forms of each x-mer were given the same index). Each nucleotide in the yeast genome was indexed according to the x-mer that begins from it. To score each x-mer occurrence, the signal around its mid position was averaged (20 bps windows). To reduce background noise, each position with signal less than 20 normalized reads was set as zero. The averaged signal for each x-mer was then calculated across all of its occurrences in all promoters and was assigned as its relative binding score. Sequence logos of the different TFs were generated based on the 10 most bound x-mers of each factor. The sequences were then aligned to the top bound motif using the Needleman–Wunsch local alignment algorithm. Each motif contributed to the probability matrix of the sequence logo based on its relative binding score.

### Data availability

Gene expression along the cell cycle: All raw data files are available in SRA under bioproject: PRJNA592756. ChEC-SEQ: All raw data files are available in SRA under bioproject PRJNA630136. Supplementary material is available at figshare: https://doi.org/10.25387/g3.13553690.

## Results

### Interspecies differences in the cell cycle program are dominated by *trans-*effects

**Figure 1:**
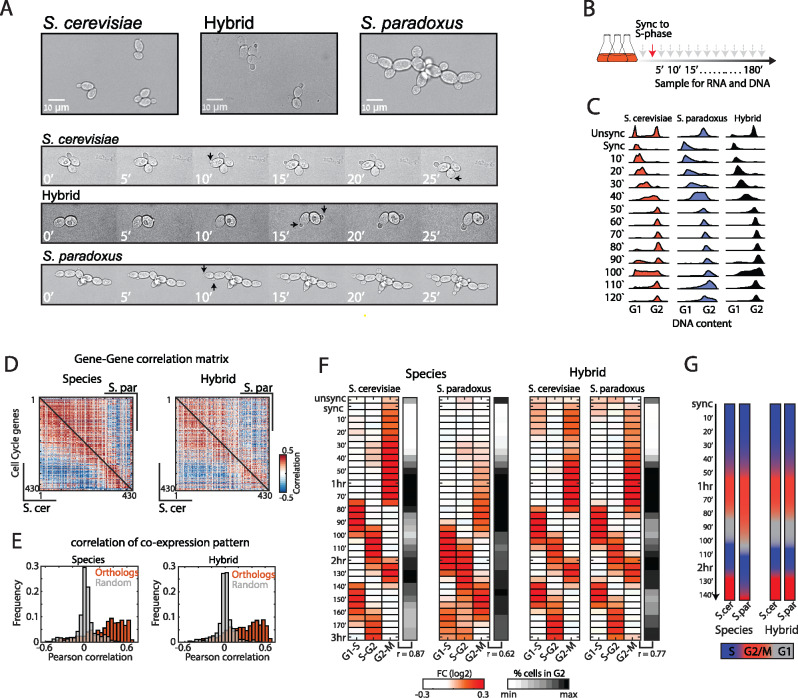
*Trans*-dominant expression program follows the growth pattern. (A) Microscopy images of *S. cerevisiae*, *S. paradoxus*, and their hybrid grown on YPD. *Saccharomyces cerevisiae* display bipolar budding and G1 daughter cell delay. *Saccharomyces paradoxus* displays unipolar and synchronized budding and remains attached after cytokinesis. The hybrid grows like *S. cerevisiae*, while exhibiting a short daughter-delay. Black arrows mark new buds. (B) Experimental layout: Cells were synchronized to early S-phase using HU, washed, and sampled for transcriptional profiling and DNA staining for 3 h in 5-min intervals. (C) Synchronized progression was monitored by measuring the DNA content using flow-cytometry (full profiles are shown in Supplementary Figure S1A). (D) Co-expression of cycling genes is conserved between species. Shown are gene–gene correlation matrices (Pearson) of periodic genes ordered by their expression peak along the cell cycle (Santos *et al.* 2015), *S. cerevisiae* is shown in the lower triangle and *S. paradoxus* in the top triangle. (E) Histograms showing the distributions of co-expression correlation coefficients between orthologous genes and between random pairs. (F) Periodicity of gene expression is synchronized with cell cycle transitions. Shown are the average fold changes of periodic genes (relative to median) classified to three groups based on expression time [defined in Santos *et al.* (2015)]. Black panel indicates percentage of cells in G2-M by DNA content profiles. Correlation values in bottom indicate the Pearson correlation coefficient between expression of the G2-M module to percent cells in G2-M by DNA content. (G) Differences in cell cycle phase dynamics: Shown is the duration of the cell cycle phases along the experiment based on (F).

We compared the growth phenotypes of the two species and their hybrid when grown in rich conditions (YPD, Figure 1A). The two species showed notable differences in growth pattern: *S. cerevisiae* (strain BY4743) cells exhibit bipolar budding, fully separate after cytokinesis, and a prolonged G1 in daughter cells as compared to their mothers (G1 daughter delay). *Saccharomyces paradoxus* (strain CBS432), on the other hand, exhibits pseudohyphal-like growth (Herbst *et al.* 2017); cells remain partially attached after cytokinesis, appear more elongated, and exhibit simultaneous budding of mother and daughter cells. We previously noted that the inter-species hybrid exhibits a growth pattern more similar to *S. cerevisiae*, yet with a shorted G1 daughter delay (Herbst *et al.* 2017). This pointed toward cell cycle regulatory differences between the two species that interact within the hybrid (Bar-Zvi *et al.* 2017).

To understand the origins of these differences, we asked whether and how the gene expression program of the two species is coordinated with their distinct cell cycle phenotypes. We grew diploids of both species and their hybrid in rich media, arrested them in early S-phase using HU and profiled their gene expression in 5-min intervals following the release from the arrest for two cell cycles (Figure 1B, Supplementary Table S2). Synchronized progression of the cells was validated by measuring their DNA content (Figure 1C and Supplementary Figure S1A). DNA profiles showed that both species and hybrid progressed uniformly following release from HU arrest, validating an efficient synchronization and release (Figure 1C). Additionally, differences in cell progression became evident in the second cycle, where *S. cerevisiae* and the hybrid started their G1 phase before *S. paradoxus* (Figure 1C, time points 80ʹ–110ʹ).

Previous studies in *S. cerevisiae* defined a set of genes showing periodic expression along the cell cycle (Spellman *et al.* 1998; Granovskaia *et al.* 2010; Santos *et al.* 2015). We examined the dynamics of these periodic genes along our time courses. The majority of them were expressed periodically in both species (72% and 65% in *S. cerevisiae* and *S. paradoxus*, respectively, Supplementary Figure S1, B–D). Co-expression patterns between orthologs of periodic genes showed high correlations both between species and within hybrid (Figure 1, D and E), confirming an overall conservation of cell cycle-regulated genes. Classifying the genes to groups based on their cell cycle expression time, revealed periodicity that was in synchrony with cell cycle phases, as defined by DNA content (Figure 1F). In particular, the G2-M delay and short G1 characteristics of *S. paradoxus* were also evident in the expression dynamics of genes activated at these phases. For example, S-phase genes followed G1 genes with a 10-min delay in *S. paradoxus*, compared to 20-min delay in *S. cerevisiae* (Figure 1, F and G and Supplementary Figure S1E). Accordingly, similarity between expression profiles of the two species followed the cell cycle phase, rather than sampling time (Supplementary Figure S1E).

The synchronization of gene expression with the cell division cycle is indicative of global dynamics guided by variations in upstream *trans-*regulators. Examining the hybrid, where both genomes are subject to the same environment, expression of periodic genes was synchronized with cell cycle progression: the two genomes followed the same dynamics (Figure 1, F and G), indicating that regulatory differences were mostly in *trans.* Furthermore, comparing the expression levels of periodic genes revealed that *trans-*effects also dominate variations in expression levels in addition to expression dynamics (Supplementary Figure S1F). Therefore, the majority of variations in cell cycle genes expression propagate from variations in upstream *trans*-acting factor(s).

### Propagating expression variation in cell cycle regulators

**Figure 2:**
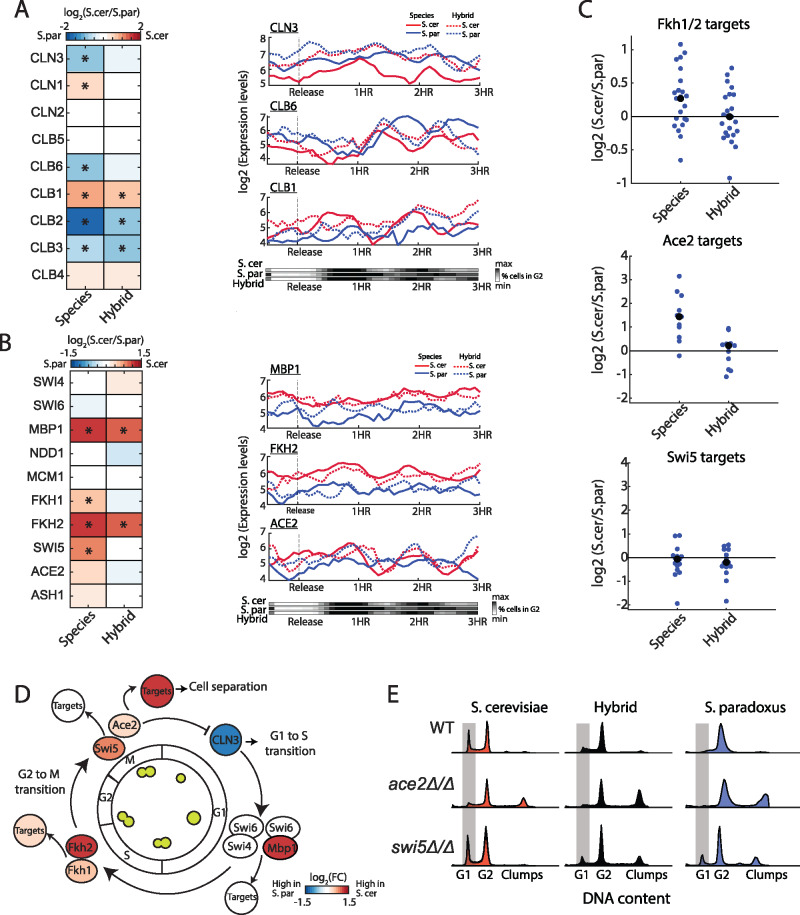
Cell cycle regulators accumulated regulatory variations that bias expression in support of the respective phenotype (A) Expression of cyclins. Left: gene expression differences between the species and between hybrid alleles. Presented fold change values are: log_2_(reads in *S. cerevisiae*/reads in *S. paradoxus*). Genes ordered by their expression time along the cell cycle. Asterisk marks significant difference in expression (corrected *P*-value < 0.05 and log_2_FC > 0.5). Right: Gene expression levels for example genes: CLN3 (early G1), CLB6 (early S), and CLB1 (G2) along the cell cycle, in synchrony with changes in DNA content (bottom panel). (B) Expression of cell cycle TFs, same as in (A). (C) log_2_ FC in TFs target genes: Fkh1/2 (Zhu *et al.* 2000), Ace2 (Voth *et al.* 2007), and Swi5 (Voth *et al.* 2007). Black dots indicate the group’s mean. Targets defined by MacIsaac *et al.* (2006) are shown in Supplementary Figure S2A. Ace2 targets are shown in detail in Supplementary Figure S2B (D) Divergence in cell cycle regulators. A scheme of cell cycle regulation, color indicates inter-species expression differences. (E) DNA contents profiles of WT, *ace2*Δ/Δ and *swi5*Δ/Δ, in both species and hybrid, taken during exponential growth in YPD. Grey area marks cells in G1 phase.

The global cell cycle expression differences result from *trans-*effects. We therefore asked whether changes in a specific cell cycle regulator or TF drives this. Cell cycle progression is driven by the periodic expression of cyclins, that together with cyclin-dependent kinases lead to the activation of phase-specific TFs (Bloom and Cross 2007). We examined the expression patterns of the three G1 cyclins (CLN1-3) which act at the G1/S transition, and the six B-type cyclins (CLB1-6) acting in later cell cycle phases (Figure 2A). Comparing expression levels revealed that six of the nine cyclins are differentially expressed between species. These inter-species differences in cyclins expression correlate with known cell cycle phenotypes when perturbing their expression: The longer duration of the G2/M phase of *S. paradoxus* for example, corresponds to the reported consequences in *S. cerevisiae* of ectopically expressing CLB3 and CLB6, or deleting CLB1(Sopko *et al.* 2006). Similarly, CLN3, a main driver of the G1/S transition (Bloom and Cross 2007), showed twofold higher expression in *S. paradoxus*, consistent with its shorter G1. Of note, variation in temporal expression of cyclins along the cell cycle was in accordance with the variation in overall cell cycle progression (Figure 2A, right panel). Examining the hybrid, we found that expression differences in G1/S cyclins (CLN3, CLN1, and CLB6) were largely suppressed, while in G2/M cyclins (CLB1-3) were largely maintained, though to a lower extent. Therefore, both *cis-* and *trans*-regulatory changes, acting in the same direction, alter the expression of cyclins and correlate with the respective cell cycle phenotype.

We next considered TFs regulating the periodic expression waves along the cell cycle and their target genes (Figure 2B, Supplementary Figure S2A). Differential expression was observed for MBP1 (log_2_ FC = 2), which acts at the G1/S transition, and for FKH1 and FKH2 (FC = 1.4 and 2, respectively), which act at S/G2. All three TFs showed higher expression in *S. cerevisiae*. For MBP1 and FKH2, this expression difference between alleles remained in the hybrid, pointing to *cis-*variation. Examining the target genes of these TFs, we find that while MBP1 targets did not show a significant change in expression (Supplementary Figure S2A), reported targets of Fkh1 and Fkh2 (Zhu *et al.* 2000) were more strongly expressed in *S. cerevisiae* (average FC = 1.25) and similarly expressed in the hybrid, as expected for *trans-*effects (Figure 2C and Supplementary Figure S2A). Among these targets are the cell cycle paralogous TFs Ace2 and Swi5, which regulate the expression of late M to early G1 genes. Similar to other Fkh2 targets, expression of SWI5 and ACE2 was higher *S. cerevisiae* (FC = 1.75 and 1.2, respectively), as was the expression of Ace2’s reported targets (Di Talia *et al.* 2009) (average FC= 2.6) but not Swi5’s (Figure 2, B and C and Supplementary Figure S2B). Additionally, Ace2’s expression showed a periodic pattern during the cell cycle *in S. cerevisiae* and the hybrid, but not in *S. paradoxus* (Figure 2B, right panel). These effects were mostly lost in the hybrid, indicating their *trans-*origin (Figure 2C).

The transcription cascade that starts in Fkh1-Fkh2 and propagates down to Ace2-Swi5 has a major effect on the cell cycle. Fkh1-Fkh2 drive the main wave of G2-M genes (“CLB2 cluster”), that includes Ace2 and Swi5, and activate progression through G2 (Zhu et al. 2000) (Figure 2D). Accordingly, deletion of both Fkh1 and Fkh2 leads to the extension of the G2 phase, decreased expression of Ace2 and Swi5’s targets and to a pseudohyphal-like phenotype (Zhu *et al.* 2000). Ace2 and Swi5 induce cytokinesis by expressing cell-separation genes required for the full separation of mother and daughter cells. Furthermore, Ace2 localizes exclusively to the daughter cell nucleus, where it inhibits the expression of CLN3, leading to an extended G1 duration in daughter cells (Laabs *et al.* 2003; Di Talia *et al.* 2009) (Figure 2D). We examined the impact the deletion of ACE2 and SWI5 would have on the cell cycle of both species and the hybrid, by measuring the DNA content of exponentially growing cells (Figure 2E). Deletion of ACE2 in *S. cerevisiae* reduced the fraction of cells found in G1 and induced clump formation, leading to a similar DNA content profile as *S. paradoxus* WT cells. Surprisingly, SWI5 deletion increased G1 duration in *S. paradoxus* and hybrid, but had no effect in *S. cerevisiae*, indicating that SWI5 has an antagonist role to Ace2 in driving G1 transition that is not apparent in *S. cerevisiae*. Together, these results suggest that interplay between the Fkh TFs propagates to Ace2 and Swi5 through both *cis-* and *trans-*effects and likely plays a key role in the phenotypic differences between these species.

### *Trans-*effects alter promoter binding specificity of Ace2 but not Swi5

In *S. cerevisiae*, expression of Swi5 is higher than in *S. paradoxus* (1.75-fold) but its targets do not show any difference, while Ace2 shows only a small difference in expression (1.2-fold) but its targets exhibit significantly higher expression. We therefore hypothesized that additional regulatory changes, besides expression, may alter the activity of Swi5 and Ace2. To test this, we profiled *in-vivo* TFs binding using ChEC-seq (Zentner *et al.* 2015) in both species and the hybrid, and examined whether binding specificities are conserved and if possible differences result from *cis-* or *trans*-regulatory variations.

**Figure 3:**
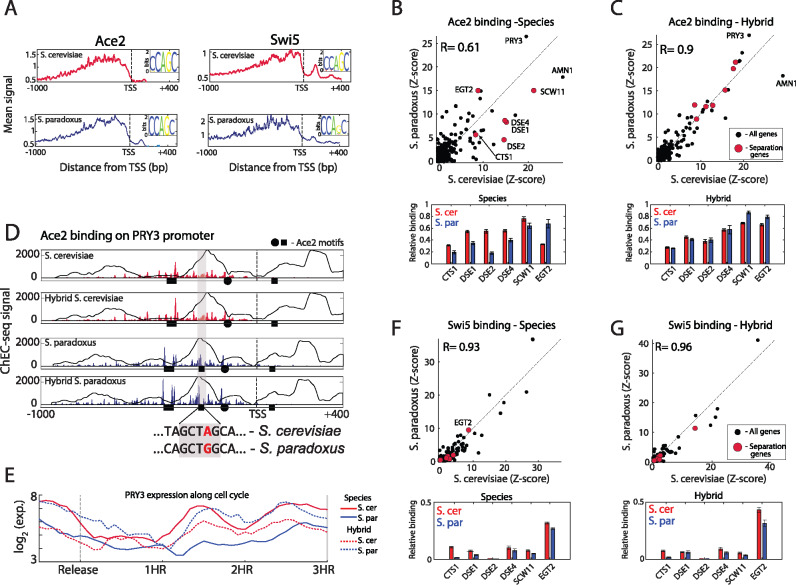
*Cis-* and *trans*-regulatory variations affect Ace2 binding (A) Meta-gene profiles of Ace2 and Swi5 in the two species. All genes were aligned by their TSS and the signal was averaged. Small panel shows the sequence logos of the DNA-motif bound by each factor based on top 10 scoring motifs. (B) ChEC-seq binding profiles of Ace2 between species. Top: plotted is Ace2 sum of signal on each promoter after Z-score standardization. Pearson correlation values of top 100 promoters are shown, genes involved in cell separation are marked in red. Bottom: Relative Ace2 binding on cell-separation genes. (C) Binding profiles of Ace2 within the hybrid, same as in (A). (D) Ace2 differentially binds PRY3 promoter. Top: Ace2 binding on PRY3 promoter, black circles and squares represent Ace2 binding motif (CCAGC) on + and − strand, respectively. Background line represents nucleosome occupancy (Tirosh *et al.* 2010). Dashed line represent the TSS (Pelechano *et al.* 2013), x-ticks are bp relative to TSS. Bottom: Sequence changes leading to loss or gain of Ace2 motif. (E) Expression of PRY3 along the cell cycle, note the *cis*-effect between hybrid alleles. (F and G) Binding profiles of Swi5 between species and within hybrid, same as in (B and C).

Binding profiles were highly correlated between repeats (*R* = 0.94–0.97 on average, Supplementary Figure S4A), and were localized upstream to the TSS (Figure 3A and Supplementary Figure S4B). In both species, Ace2 and Swi5 showed strong binding to their preferred *in-vitro* binding motif (de Boer and Hughes 2012) (CCAGC, Figure 3A). We next examined the promoter binding specificities of the TFs. Comparing Ace2 binding between species, we find that the overall promoter selection has diverged (Figure 3B, top; promoter selection correlation = 0.61). More specifically, promoters of cell-separation genes (CTS1, DSE1, DSE2, DSE4, and SCW11), which are the main targets of Ace2 (Weiss 2012), were bound to a higher level in *S. cerevisiae* than in *S. paradoxus* (Figure 3B, bottom; 1.8-fold higher signal on average). Notably, EGT2, the only Swi5-regulated cell-separation gene (Weiss 2012), did not conform to this general behavior and showed higher binding signal in *S. paradoxus*. We next asked whether Ace2 binding differences may result from differences in Ace2 expression levels or protein sequence. We first reduced ACE2 expression in a diploid *S. cerevisiae* by deleting one of its alleles. This, however, had little effect on its binding specificity (Supplementary Figure S3D; *R* = 0.94). Similarly, binding profiles of the two Ace2 orthologs within the hybrid were highly similar (Supplementary Figure S3C; *R* = 0.99). Therefore, differences in Ace2 binding specificity in the two species are not due to differences in Ace2 expression level or protein sequence.

Variations in Ace2 binding could result from *cis*-mutations in its target genes promoters, or from variations in a *trans-*acting factor. To examine this, we compared Ace2 binding to the two genomes within the hybrid (Figure 3C, top). The majority of differences in Ace2 promoter binding specificities between the species were suppressed within the hybrid, indicating on *trans-*acting variation (Figure 3C;*R* = 0.9). In particular, cell-separation alleles of both *S. cerevisiae* and *S. paradoxus* were bound by Ace2 to a similar extent (Figure 3C, bottom). *Cis-*effects, on the other hand, were observed in a small number of genes; PRY3, for example, a cell wall associated protein, was bound to a higher level (1.4-fold) in *S. paradoxus allele*, while AMN1, a modulator of cell separation and mitotic exit, was more strongly bound (1.5-fold) in *S. cerevisiae* allele(Figure 3D and Supplementary Figure S3E). In both cases, one Ace2 binding site was absent from the promoter showing lower binding, either due to a single nucleotide polymorphism (SNP, in PRY3) or to a single base-pair deletion (in AMN1). Furthermore, expression of the respective alleles varied in *cis* (within the hybrid), supporting the functionality of this gain (or loss) of binding site (Figure 3E). We also noted reduced Ace2 binding in *S. paradoxus* to the CLN3 promoter, which was associated with the loss of an Ace2 binding site due to an SNP (Supplementary Figure S3F). This site was previously reported to affect the duration of G1 phase *in S. cerevisiae* (Di Talia *et al.* 2009). We hypothesized that loss of this site in *S. paradoxus* may account for its short G1. Mutating this binding site to match the *S. paradoxus* allele led to a significant, albeit small, reduction in G1 duration in *S. cerevisiae* as measured both by DNA staining and live microscopy (5%, Supplementary Figure S3, G and H). Therefore this *cis*-variation in CLN3 likely contributes to the reduced G1 duration of *S. paradoxus*, yet cannot fully account for it on its own (50% reduction compared to *S. cerevisiae*; Herbst *et al.* 2017). These results show that Ace2 binding differences between the species are mostly due to variations in *trans*, while *cis-*variations play a smaller role.

We next asked whether such *trans*-variations also affect the binding of Ace2’s paralog, Swi5. In contrast to Ace2, Swi5 binding specificities were largely conserved between the species and within the hybrid (*R* = 0.93, 0.96, respectively, Figure 3, F and G, top). Swi5 binding to cell-separation promoters was significantly weaker than that of Ace2 (Figure 3, F and G, bottom). Together, these results show that *trans*-variations affect Ace2 promoter specificities, without changing its motif preference and without affecting its paralog, Swi5.

### Fkh1 and Fkh2 bind *trans-*effected Ace2 targets

**Figure 4:**
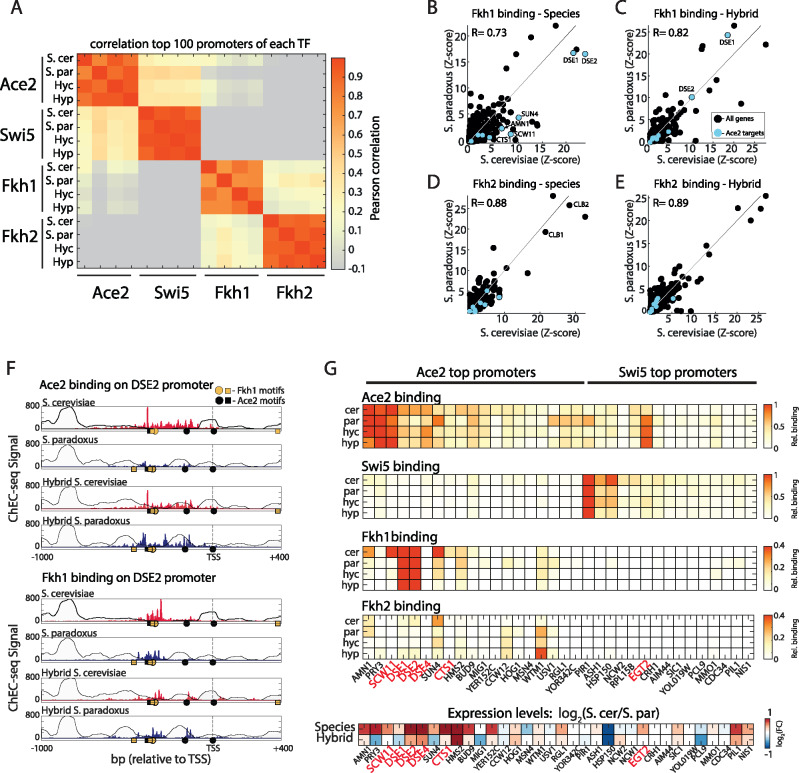
Divergence in binding of Fkh1 and Fkh2 on Ace2 targets (A) Pearson correlation matrix between the top 100 promoters of Ace2, Swi5, Fkh1, and Fkh2. Hyc and Hyp refer to Hybrid *S. cerevisiae* genome and *S. paradoxus* genome, respectively. Note that Fkh1 shows low-positive correlation with *S. cerevisiae* Ace2. (B–E) Binding profiles of Fkh1 and Fkh2 between species and within the hybrid, same as Figure 3B, marked in blue are Ace2 targets (Voth *et al.* 2007). (F) Binding of Ace2 and Fkh1 on the cell-separation gene DSE2. Circles and squares represent Ace2 binding motif (CCAGC, black), and Fkh1 binding motif (GTAAACA, yellow) on + and − strand, respectively. Note *trans*-effects both in Ace2 and in Fkh1. (G) Binding on the top promoters of Ace2 and Swi5. Top: Shown are relative sum of signal on promoters for each factor in the species and hybrid. Note overlap of Fkh1 and Fkh2 with Ace2 promoters. Bottom: Differences in expression levels. Cell-separation genes are marked in red.

Fkh1 and Fkh2, the upstream activators of Ace2 and Swi5, were previously shown to bind Ace2-specific promoters and block their activation by Swi5 (Voth *et al.* 2007). We hypothesized that they may also regulate Ace2 activity by promoting its binding to specific promoters and lead to the *trans-*variations in Ace2 binding. We therefore profiled Fkh1 and Fkh2 binding in both species and the hybrid. Binding profiles highly correlated between repeats and were localized upstream of the TSS (Supplementary Figure S4, A and B), and both TFs showed strong binding to their known consensus motif (de Boer and Hughes 2012, Supplementary Figure S4, C and D). Examining the TFs’ promoter selection revealed that while Fkh1 and Fkh2 bind a distinct set of promoters than Ace2 or Swi5, Fkh1 and Ace2 showed a low but significant correlation in promoter selection in *S. cerevisiae* (*R* = 0.25) but not in *S. paradoxus* (*R* = 0.02, Figure 4A). Specifically, Ace2 targets were strongly bound by Fkh1, and to lesser extent by Fkh2 (Figure 4, B–E). Comparing the species, Fkh1 showed lower binding to Ace2 targets in *S. paradoxus* relative to *S. cerevisiae* (Figure 4B). When examining the hybrid, differences in Fkh1 binding on Ace2 targets were largely buffered (Figure 4C), implying that Fkh1 is also affected by *trans*-regulation in the levels of binding, though to a lower extent than Ace2. The promoter of DSE2, for example, was more strongly bound by both Ace2 and Fkh1 in *S. cerevisiae* than in *S. paradoxus*, and this difference was buffered in the hybrid (Figure 4F). These results imply that Ace2 and Fkh1 cooperate in *S. cerevisiae* to activate cell-separation genes. To examine this, we asked whether binding differences correspond with expression differences. Indeed, Ace2 targets that are also bound by Fkh1 and Fkh2, showed higher expression in *S. cerevisiae* than in *S. paradoxus*, which was mostly buffered in the hybrid (Figure 4G). Overall, these results suggest a cooperation between Ace2 and Fkh1 (and to lower extent Fkh2) at the level of binding which is perturbed in *S. paradoxus.* This cooperative binding appears mostly on promoters of cell-separation genes, which are differentially expressed between the species, mostly due to *trans*-regulatory variation.

### After deletion of the Fkh TFs, Ace2 binding in *S. cerevisiae* mimics *S. paradoxus*

**Figure 5:**
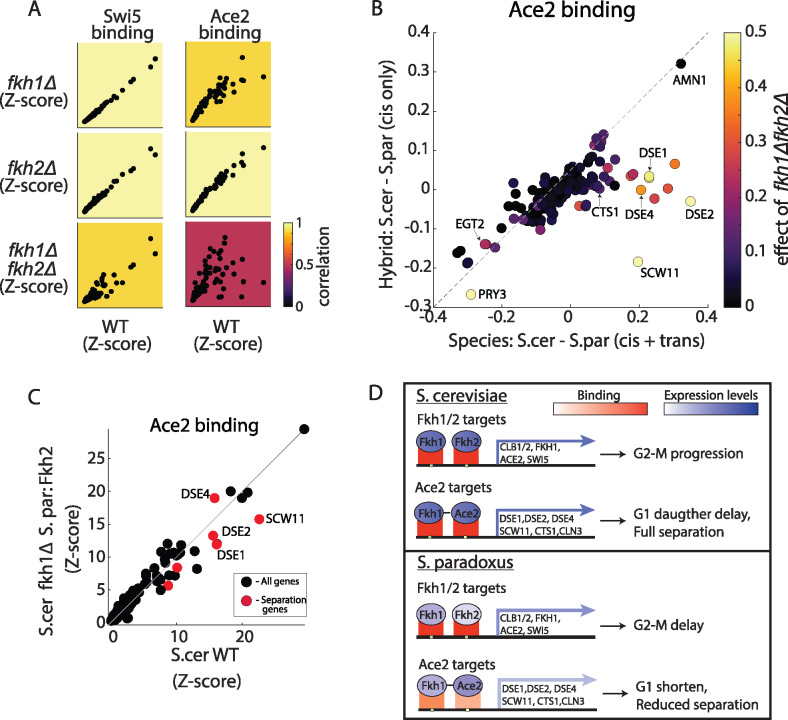
Fkh1 and Fkh2 directly mediate Ace2 binding to cell-separation genes (A) Ace2 binding is mediated by Fkh1 and Fkh2. Shown are normalized sum of signal on promoters for Swi5 and Ace2 in WT *S. cerevisiae* compared to *fkh1*Δ, *fkh2*Δ, and *fkh1*Δ*fkh2*Δ. Color represents correlation of top 100 promoters to WT. (B) *Cis-* and *trans*-effects on Ace2 binding. Scatter plot of relative Ace2 binding differences between species (*x*-axis) and between hybrid alleles (*y*-axis). Color represents the differences in Ace2 binding between *S. cerevisiae* WT to *fkh1*Δ*fkh2*Δ. Note that cell-separation genes show a strong *trans*-effect and are strongly affected by *fkh1*Δ*fkh2*Δ. (C) *S. paradoxus*’ FKH2 is sufficient to restore Ace2 binding in *S. cerevisiae*. Scatter plots of normalized sum of signal on promoters of Ace2 in *S. cerevisiae* WT vs. *S. cerevisiae fkh1*Δ expressing *S. paradoxus* FKH2. Red dots represent cell-separation genes. (D) Scheme for proposed mechanism driving cell cycle variation: in *S. cerevisiae*, higher expression of Fkh1 and Fkh2 leads to higher expression of target genes and faster G2-M transition. Fkh1 (and Fkh2, to lower extent) binds Ace2 target genes and recruits Ace2, leading to higher expression of target genes, G1 daughter-cell delay and cell separation. In *S. paradoxus*, lower expression of Fkh1 and Fkh2 leads to reduced expression of target genes (including Fkh1 itself and Ace2) and longer G2. Downstream, Fkh1 exhibits reduced binding to Ace2 targets (*trans*-effect) leading to reduced binding of Ace2, lower expression of target genes, shorter G1-phase and reduced separation.

We hypothesize that Fkh1 and Fkh2 localize to cell-separation promoters and recruit Ace2 in *S. cerevisiae* but not in *S. paradoxus.* To test whether Fkh1 and Fkh2 recruit Ace2, we examined Ace2 and Swi5 binding in *S. cerevisiae* cells deleted of FKH1, FKH2, or both. Deleting either FKH1 or FKH2 had little effect on binding of Ace2 and Swi5 (*R* = 0.92–0.99, Figure 5A). Swi5 binding was also largely invariant to the deletion of both factors (*R* = 0.93), yet binding of Ace2 to its top promoters was largely suppressed (*R* = 0.53, Figure 5A). More specifically, deletion of both FKH1 and FKH2 affected Ace2 binding primarily in promoters showing high *trans*-variations toward *S. cerevisiae* (Figure 5B). Since these double-deletion cells exhibit pseudohyphal-like growth, we controlled for nonspecific effects of the pseudohyphal phenotype by over-expression of CLB2 in this background (Hollenhorst *et al.* 2000). While this overexpression retrieved the yeast growth phenotype, Ace2 did not regain binding to cell-separation promoters (Supplementary Figure S5, A–C). Together, these results suggest that Fkh1 and Fkh2 directly regulate the recruitment of Ace2 to promoters, and that this recruitment is perturbed in *S. paradoxus*.

We reasoned that the lower expression levels of FKH1 and FKH2 in *S. paradoxus* could explain the reduced binding of Ace2 to cell-separation promoters in this species. FKH2 activates the expression of FKH1 and shows twofold lower expression in *S. paradoxus* as a result of a *cis-*effect. We therefore swapped the *S. cerevisiae* FKH2 allele (promoter plus ORF) with that of *S. paradoxus*. While this swapped strain indeed showed lower expression of FKH2 (Supplementary Figure S5B), it was sufficient to restore Ace2 binding to FKH1/2-deleted cells (Figure 5C). In addition, this allele-swap was sufficient to fully suppress the pseudohyphal phenotype of the double FKH1/2-deleted *S. cerevisiae* strain (Supplementary Figure S5C). Therefore, an additional layer of variations exists, whereby low levels of FKH2 are sufficient to recruit Ace2 binding in *S. cerevisiae* but not sufficient for its recruitment in *S. paradoxus*. These variations likely result from *trans* through additional signaling or other unknown factor that affect the interactions between Ace2 and the Fkh TFs. Taken together, our results reveal inter-species regulatory variation acting both in expression levels and in TFs binding. This variation originates from multiple *cis-* and *trans*-regulatory changes, which propagate through the cellular network and alter cell cycle progression.

## Discussion

A major challenge in the study of regulatory evolution is to link variations in phenotypes to molecular differences in the underlying regulatory network. Here, we examine this by comparing budding yeast species that express largely the same set of genes and exhibit similar growth requirements, yet show marked differences in cell cycle dynamics when grown in the same environment. These differences are evident from the shorter G1 of *S. paradoxus*, its prolonged G2 and lack of cell separation in comparison to *S. cerevisiae*. The cell cycle regulatory network has been extensively studied, providing a tractable model for systematic analysis of regulators and target genes. By measuring both the cell cycle transcriptome in high resolution, and TFs binding specificities in both species and the hybrid, we provide a detailed map of the regulatory variation underlying this phenotypic divergence.

The cell cycle program requires the temporal activation of a large number of genes, with oscillating waves of expression that are driven by cell cycle-specific TFs. Therefore, phenotypic variation in cell cycle progression is likely to propagate throughout the network. Indeed, our analysis revealed that variation in periodic genes was dominated by propagating *trans-*effects. Similar results were previously shown for meiosis, in which initial *trans*-effects dominate heterochronic differences between the two species and their hybrid (Swain Lenz *et al.* 2014). Our analysis poised Ace2, Fkh1 and Fkh2 as principal factors driving this divergence. We noted that expression of the TFs FKH1 and FKH2 is lower in *S. paradoxus* as is the expression of the targets of their downstream TFs, Ace2 and Swi5. Furthermore, we found that regulatory changes in Fkh1 and Fkh2 not only affect ACE2 and SWI5 expression, but also the recruitment of Ace2 to cell-separation genes, resulting in stronger binding of Ace2 in *S. cerevisiae* (Figure 5E). These changes correlate with the partial cell separation and absence of G1 daughter delay in *S. paradoxus*. In addition, deletion of either Ace2 or the Fkh TFs in *S. cerevisiae* leads to similar changes in cell cycle phases as seen between the species. These binding differences of Ace2, and to lower extent Fkh1, were attributed mostly to *trans*-regulatory variation, rather than changes in protein sequence or *cis-*variations in target genes. These results illustrate how substantial differences in TFs binding and TF cooperation can diverge between close species and have a direct impact on phenotype.

We did not track the source variation that drives these propagating *trans*-effects. These may come from other interacting pathways. The *S. paradoxus* strain used in this study (CBS432) exhibits pseudohyphal-like growth, a phenotype that typically occurs in yeast deprived of nitrogen and includes changes in cell cycle and morphology (Cullen and Sprague 2012). It is therefore plausible that variations in pseudohyphal growth regulators play a role. Supporting this, Fkh1, Fkh2, and Ace2 have all been previously linked to the regulatory network controlling the transition to pseudohyphal growth (King and Butler 1998; Zhu *et al.* 2000). There is large variation even between strains of the same species in the extant and ability to induce pseudohyphal growth; Different *S. paradoxus* strains show differences in growth patterns and morphology (Roop and Brem 2013). In addition, the *S. cerevisiae* strain (BY4743) used in this study carries several loss-of-function mutations making it incapable of pseudohyphal growth even under nitrogen starvation (Liu *et al.* 1996). Therefore, our results might represent strain-specific variations rather than species-specific. However, the fact that the hybrid exhibits a growth pattern that is similar to *S. cerevisiae*, even though it contains functional alleles from *S. paradoxus*, implies that additional variation exists. In addition, the hybrid exhibits cell cycle phenotypes attributed to each of its parents, with lack of G1-delay as in *S. paradoxus*, and shorter G2 as in *S. cerevisiae*, supporting the view of complex regulatory variation. Notably, the hybrid parent-like behavior, rather than mid-parent behavior in these cell cycle phenotypes, implies that in each phenotype one parental-version is dominant over the other, as might be expected from a *trans*-varied expression module (Krieger *et al.* 2020).

Our work shows how regulatory variations propagate through the cellular network and alters the expression of numerous genes. We uncovered additional regulatory variation in the level of TFs binding, in which changes in cooperation between TFs leads to changes in TFs promoter binding specificity. These regulatory variations accumulate and propagate through the cell cycle regulatory network in support of the different cell cycle phases phenotypes. It is likely that additional variations, in either pseudohyphal regulation or other cellular processes interact and drive this phenotypic divergence. This falls in line with the omnigenic model of inheritance (Liu *et al.* 2019), which predicts that variation in complex traits is by and large shaped by multiple *trans*-acting variation, due to the high connectivity within the transcriptional network. This connectivity expands the space of variations propagating to modulate the principal phenotypic-relevant pathways.
